# Prior Exposure to Uninfected Mosquitoes Enhances Mortality in Naturally-Transmitted West Nile Virus Infection

**DOI:** 10.1371/journal.pone.0001171

**Published:** 2007-11-14

**Authors:** Bradley S. Schneider, Charles E. McGee, Jeffrey M. Jordan, Heather L. Stevenson, Lynn Soong, Stephen Higgs

**Affiliations:** 1 Department of Pathology, Center for Biodefense and Emerging Infectious Diseases, University of Texas Medical Branch, Galveston, Texas, United States of America; 2 Department of Microbiology and Immunology, University of Texas Medical Branch, Galveston, Texas, United States of America; 3 Pasteur Institute, Paris, France; University of Liverpool, United Kingdom

## Abstract

**Background:**

The global emergence of West Nile virus (WNV) has highlighted the importance of mosquito-borne viruses. These are inoculated in vector saliva into the vertebrate skin and circulatory system. Arthropod-borne (arbo)viruses such as WNV are transmitted to vertebrates as an infectious mosquito probes the skin for blood, depositing the virus and saliva into the skin and circulation. Growing evidence has demonstrated that arthropod, and recently mosquito, saliva can have a profound effect on pathogen transmission efficiency, pathogenesis, and disease course. A potentially important aspect of natural infections that has been ignored is that in nature vertebrates are typically exposed to the feeding of uninfected mosquitoes prior to the mosquito that transmits WNV. The possibility that pre-exposure to mosquito saliva might modulate WNV infection was explored.

**Principal Findings:**

Here we report that sensitization to mosquito saliva exacerbates viral infection. Prior exposure of mice to mosquito feeding resulted in increased mortality following WNV infection. This aggravated disease course was associated with enhanced early viral replication, increased interleukin-10 expression, and elevated influx of WNV-susceptible cell types to the inoculation site. This exacerbated disease course was mimicked by passive transfer of mosquito-sensitized serum.

**Significance:**

This is the first report that sensitization to arthropod saliva can exacerbate arthropod-borne infection, contrary to previous studies with parasite and bacteria infections. This research suggests that in addition to the seroreactivity of the host to virus, it is important to take into account the immune response to vector feeding.

## Introduction

West Nile virus (WNV) is a positive sense, single-stranded RNA virus (family *Flaviviridae*) that emerged globally following the appearance of a more neurotropic subtype [1]. Recently outbreaks of WNV infection have occurred in the Middle East, Europe, Africa, South America, and North America. Since its introduction into the United States in 1999, WNV has spread rapidly, leading to >19,000 diagnosed human cases [2] and an estimated 750,000 undiagnosed infections [3]. Clinically, WNV infection typically presents as a mild febrile illness, with occasional progression to fatal neuroinvasive disease [4]. The virus is transmitted to vertebrates usually as an infectious mosquito probes the skin for blood, depositing saliva and virus in extravascular tissue and circulatory system [5]. Accumulating evidence has demonstrated that the arthropod saliva, which carries WNV into the vertebrate, is not simply a transport medium, but can have a profound effect on pathogen transmission efficiency, pathogenesis, and disease course [6]–[12]. Following peripheral inoculation, WNV has limited replication in the skin; and within 24 h of deposition, infected dendritic cells (DCs), particularly Langerhan cells (LCs), migrate from the epidermis to local lymph nodes (LN) [13]. The role of these cells in flavivirus infection may be pleiotropic, as their migration to draining LN promotes antigen presentation, whilst simultaneously facilitating viral dissemination. There is a close relationship between early peripheral virus burden and proclivity towards neuroinvasion and death [14]. Numerous studies have demonstrated that early events, such as timing and magnitude of viremia, integrity of innate immune response, activity of type I interferons (IFN), and early replication efficiency of virus, are acutely important in the survival of the host [4]. Early shifts in the host immune response may thus lead to significant differences in disease course.

Exposure to uninfected mosquitoes is common, and both humans and rodents develop an immune response to mosquito salivary proteins, characterized by IgE/IgG reactivity, immediate wheal and flare reaction, and/or delayed type hypersensitivity [15]. Previous research demonstrated that mice pre-exposed to the salivary gland extract (SGE) of sand flies followed by infection with *Leishmania major* in the presence of SGE have a five-fold increase in infiltrating leukocytes in the skin, augmented IFN-γ, decreased interleukin (IL)-4, and, remarkably, are protected against the severe disease [12], [16]. Pre-exposed hosts also have a reduced *Plasmodium yoelii* burden [17]. In response to mosquito salivary antigens, however, sensitized mice also show a significantly increased lymphocyte proliferation response, but, conversely, have an enhanced IL-4 and decreased IFN-γ production, suggesting that a T_H_2 immune response predominates despite the development of the delayed skin reaction [15].

The effects of pre-exposure to mosquito saliva upon viral infection have not been defined. Thus, in this study we investigated the potential for an immune response directed against mosquito salivary proteins to have a protective or antagonistic effect on naturally transmitted arboviral infection.

## Results

**Figure 1:**
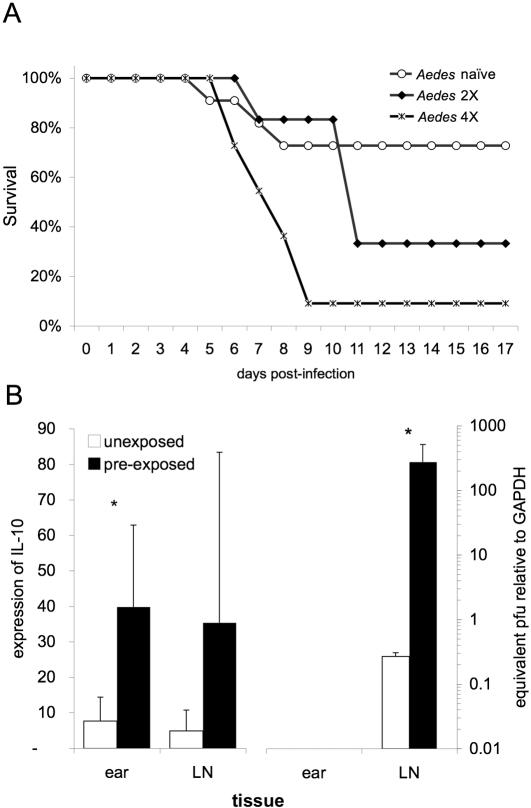
Altered WNV pathogenesis mediated by prior host exposure to uninfected mosquito feeding. (A) Survival of BALB/c mice infected with WNV by the bite of a single *Ae. aegypti* mosquito. Mosquitoes were infected 7 days prior to feeding via intrathoracic inoculation and the infection of each mosquito was confirmed immediately after feeding via real-time RT-PCR. Prior to infection, mice were exposed to 15–20 mosquitoes once weekly for a total of 0, 2, or 4 times. Data are the pooled results of 3 separate replicates of this experiment (n≥5 mice/group/replicate). Difference in survival is significant (Fisher's exact test; *p*<0.05). (B) Levels of IL-10 (left) and WNV (right) RNA in mosquito feed site (ear) and draining LN at 36 h post-infection as detected by real-time RT-PCR and normalized to glyceraldehyde-3-phosphate dehydrogenase (GAPDH) expression. * indicates a *p*-value of less than 0.05.

Prior exposure of BALB/c mice to *Aedes aegypti* feeding (two or four exposures to ∼17 uninfected mosquitoes over the course of one month) resulted in increased mortality due to mosquito-transmitted WNV infection (Fig. 1A). More intense pre-exposure corresponded to enhanced mortality: with 68% and 91% mortality observed in groups previously exposed two and four times to uninfected mosquitoes, respectively, compared to 27% mortality in unexposed mice (*p*<0.01).

Subsequent experiments investigated the mechanism of this divergence in disease severity. The memory response to mosquito proteins could skew the immune response in a manner that compromises early anti-viral defenses. The microenvironment at the site of viral invasion and initial replication, including cytokines and immune cells present can influence the early orchestration of the response and priming of T cells. Previous research related to mosquito allergy demonstrated a T_H_1 to T_H_2 shift following sensitization to *Ae. aegypti* [15]. To evaluate whether cytokine expression differed between groups, total RNA was isolated from inoculation site and draining LN for quantifying the relative levels of IL-2, IL-4, IL-10, IL-12p40, IFN-β, and IFN-γ via real-time RT-PCR [9].

In three separate experiments, IL-10 expression was consistently elevated in the dermis and draining LNs of sensitized mice as compared to naïve mice at 36 h post-infection (*p* = 0.04)(Fig. 1B). This immuno-regulatory cytokine is multifunctional. In relation to viral infections, IL-10 may create favorable conditions for viral replication by disarming the innate and adaptive responses. In human monocytes, IL-10 and IFN-γ antagonize the function of each other [18]. IL-10 also down regulates MHC class II antigen expression by monocytes and inhibits antigen presentation by several types of antigen-presenting cells (APC), including epidermal LCs [19], [20]. The presence of viruses whose genomes code for IL-10 homologs, demonstrate that there is an evolutionary advantage to enhanced IL-10 levels for an invading virus [21], [22]. Studies with peripheral blood leukocytes found that during the early phase of infection with dengue virus, increased IL-10 production induces lasting T cell inactivation and decreases the control of virus infection [23]. Consequently, the interaction of immunosuppressive IL-10-producing cells with T cells early during WNV infection may result in the loss of T-cell responsiveness and facilitate an enhancement of viral replication and a blunted adaptive immune response. Thus, notwithstanding further effects of prior exposure to mosquitoes, heightened IL-10 production associated with an anti-mosquito immune response may account for the altered course of infection.

No clear pattern was observed with T_H_1 cytokines however, expression of IL-4 was 50-times higher in LNs of sensitized mice at 36 h post-infection. Although this difference was not statistically significant, the consistency of the increased expression in mosquito-sensitized mice suggested a trend towards an elevated T_H_2 response in pre-exposed mice. The enhancement of IL-4 following *Ae. aegypti* exposure parallels that observed with previous research [15].

**Figure 2:**
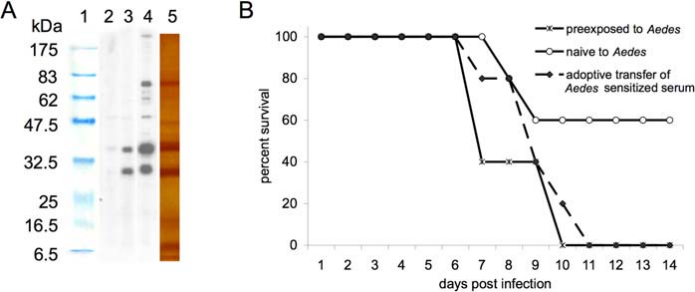
Passive transfer of mosquito saliva-sensitized serum affects disease coursed. (A) Western blot analysis of *Ae. aegypti* salivary glands; sera from unimmunized BALB/c mice (lane 2), mice exposed twice to mosquito feeding (lane 3), and mice exposed 4 times to mosquito feeding (lane 4). Protein standards and SGE proteins visualized by silver stain are shown in lane 1 and 5, respectively. (B) Survival following WNV infection in naïve, pre-exposed (4 times), and passively immunized BALB/c mice (via intraperitoneal inoculation of 200 µL of mosquito-sensitized serum 12 h prior to infection). Mortality was significantly different between naïve and passively immunized mice (p<0.05).

Passive transfer of serum from exposed mice to naïve mice resulted in elevated mortality (Fig. 2B), characteristic of the group previously exposed to mosquito feeding, thus implicating the humoral immune response in the deviation of pathogenesis in mosquito-sensitized mice (Methods S1). This does not preclude the possibility that the cellular response is also important. Anti-salivary protein antibodies were readily detected in pre-exposed mice (Fig. 2A). Western blot analysis of the serum (Methods S2) showed six major bands recognized by the serum of *Ae. aegypti*-exposed mice, with increased reactivity observed in mice exposed four times to mosquito feeding (Fig. 2A).

It has been suggested that the presence of anti-saliva antibodies in blood might conceivably impair the feeding efficiency of infected mosquitoes by inhibiting salivary protein function [24]. This could necessitate greater probing activity, thereby increasing the titers of WNV inoculated into the skin. Using Anopheline mosquitoes, one study demonstrated that the suppression of the mosquito salivary protein apyrase activity was associated with decreased competence locating blood, increased probing time, and presumably increased inoculation of malaria parasites [24]. In the current study, there was no significant difference in the number of blood-engorged mosquitoes recovered from naïve versus pre-exposed mice. Additionally, treatment of *Ae. aegypti* SGE with serum from sensitized mice caused no reduction in apyrase activity as judged by an *in vitro* biochemical assay that measures the release of phosphate from adenosine triphosphate (Methods S3; data not shown). The titer of virus inoculated into mice was estimated by dissecting mosquito salivary glands immediately following feeding on mosquito immune and non-immune mice, and by comparing with the titers in salivary glands of unfed mosquitoes. No significant difference was observed between these groups (data not shown). Therefore, the observed effect of sensitization to mosquito saliva appears not to be due to a direct effect on the efficacy of mosquito feeding, but rather to a response subsequent to probing.

**Figure 3:**
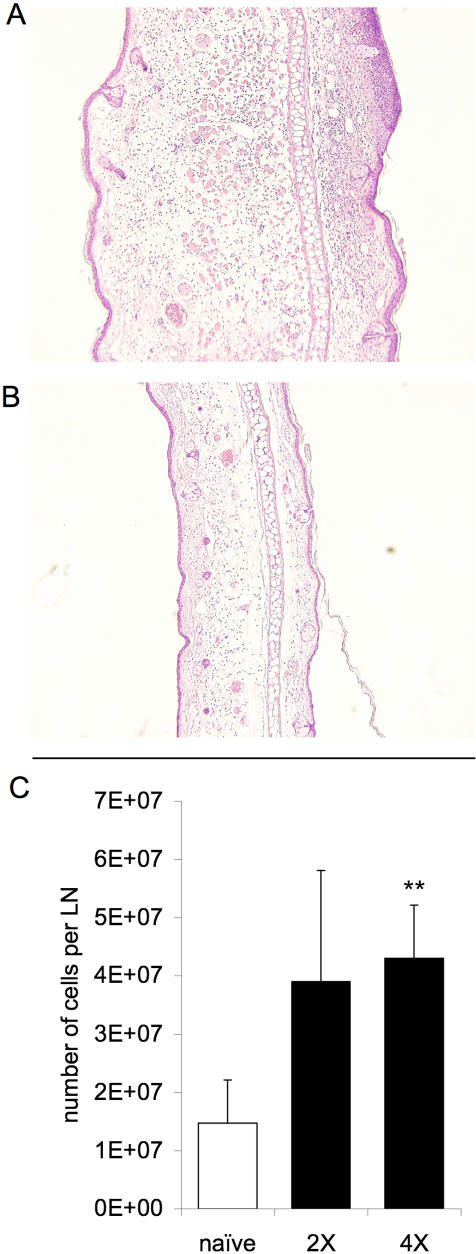
Cell influx into early sites of WNV replication. Dermal site of *Ae. aegypti* feeding 36 h post-exposure in mosquito-sensitized (A) and mosquito-naïve (B) mice. Sections are representative of observations (3 mice per group). (C) Number of cells per retromaxillar LN 36 h post infection initiated via the feeding of a single infected mosquito on the pinna of the ear (n = 6); ** represents a significant difference compared to mice previously naïve to *Ae. aegypti* by paired t-tests among each replicate (*p*<0.01).

**Figure 4:**
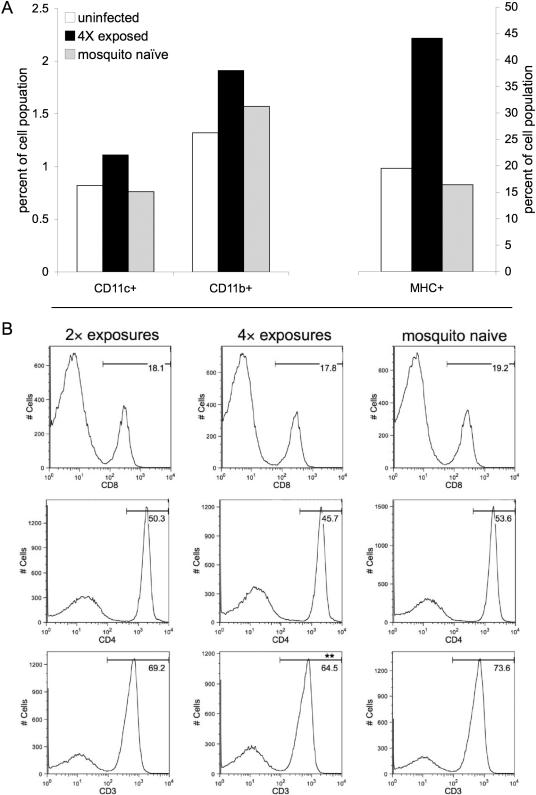
Identification of differential cell influx into mosquito feed site and draining LN. (A) Proportion of the skin cell population composed of CD11c^+^, CD11b^+^, and MHC class II^+^ cells 36 h post infection as assessed by flow cytometry. Mice were pre-exposed or not to *Ae. aegypti* feeding prior to infection and a control uninfected group was examined as well to assess baseline cell proportions. (B) Histograms illustrating the quantity of LN cells with the indicated intensity of CD8, CD4, or CD3 staining at 36 h post infection; the x-axis corresponds to the cell surface expression level the indicated membrane protein. Percent of CD8^+^, CD4^+^, or CD3^+^ cells in the LN draining the site of virus inoculation is specified. Asterisks signify a statistically significant difference compared to mice previously naïve to *Ae. aegypti* via paired t-tests among each replicate: ** *p*<0.01. Groups of mice (n = 3) were exposed 0, 2, or 4 times to mosquito feeding prior to the feeding of a WNV-infected mosquito. Results consist of a pool of cells from three individual mice and are representative of a total of three replicates.

Histological analysis of the skin and draining LN at 24 and 48 h post-infectious bite revealed a transient, mild inflammatory reaction in naïve mice, in contrast to significant cellular infiltrate and a >200% increase in tissue size in pre-exposed mice. The edema at the feeding site in sensitized mice was characterized by mononuclear (plasma and histocytic) cell and neutrophil infiltrate (Fig. 3A,B). As few as two pre-exposures to uninfected mosquitoes were sufficient to stimulate marked LN expansion at 36 h post-exposure to infected mosquito feeding; with 3.9±0.9×10^7^ and 4.3±0.9×10^7^ cells per LN for mice with two and four times pre-exposures, respectively, as compared to 1.5±0.7×10^7^ cells per LN in naïve mice exposed to an infected mosquito (Fig. 3C; *p*<0.01). One possible explanation for the increased severity of WNV infection in sensitized mice is that the immune response to salivary proteins promotes the recruitment of susceptible cell types to the inoculation site and consequently increases opportunity for virions to infect target cells. To investigate this hypothesis, cells recovered from the skin and draining LN after mosquito inoculation of WNV were stained with appropriate antibodies and analyzed by flow cytometry [16]. All groups of mice exposed to WNV displayed an inflammatory response to viral replication in the form of cell influx into the effected tissues. However, in mice pre-exposed to mosquito feeding, the inflammatory response was significantly elevated in comparison to non-sensitized mice. Following WNV infection, the frequency of MHC class II-expressing skin cells was more than double in mice sensitized to *Ae. aegypti* feeding (Fig. 4A). MHC class II molecules are predominantly present on DCs, monocytes, and macrophages, cells highly susceptible to WNV infection [25], [26]. Similarly, the frequencies of CD11c^+^ and CD11b^+^ cells, mostly APCs and granulocytic cells, were also higher after infection in the skin of mosquito-pre-exposed mice (Fig. 4A). Predictably, influx of these cell types was also elevated in the draining LNs of mice with prior exposure to mosquitoes. (*p*<0.05)

Analogous to observations in the skin, determination of the abundance of subpopulations within LNs revealed a consistent pattern: following WNV infection via mosquitoes, mice that were pre-exposed to naïve mosquitoes displayed an enhanced leukocyte level, including CD11c^+^ and CD11b^+^ cells, concurrent with reduced lymphocyte populations, as revealed by the lowered CD3^+^ cell percentage (Fig. 4B; *p*<0.01). In mice sensitized to mosquito feeding prior to WNV infection, CD3^+^ cells comprised 69.2% of LN cells in mice exposed twice, and 64.5% of LN cells in mice exposed four times at 36 h post-infection, as compared to 73.6% of cells in non-sensitized mice illustrating that previous exposure results in a dose dependent decrease in the CD3^+^ population (Fig. 4B). With respect to specific T cell populations, the CD4^+^ cell density was consistently lower in mosquito-sensitized mice, although this difference was not statistically significant; cells expressing CD4 on their surface were 53.6% of the cell population in naïve mice compared to 50.3% and 45.7% in mice previously exposed to mosquitoes twice and four times, respectively. CD8-expressing T cell density was marginally affected by prior exposure to mosquito feeding, which accounted for 19.2%, 18.1%, and 17.8% of the LN population in naïve, twice and four times exposed, respectively (Fig. 4B). The observed trend in CD4^+^ cell numbers resulting from prior exposure to mosquito feeding could help to explain the divergence in WNV infection, although the elevated influx of leukocytes and CD3^−^ cells likely play a role in this proportional decline. A recent study with WNV [27] illustrated the important role that CD4^+^ T cells play in controlling infection. Suppression or deficiency of this subpopulation during WNV infection in mice resulted in prolonged central nervous system infection and uniform lethality. Additionally, mice lacking CD4^+^ T cells had reduced IgG production and, later in infection, compromised WNV-specific CD8^+^ T cell activation and trafficking to the CNS [27]. Mice lacking CD8^+^ T cells have higher CNS viral burdens and increased mortality rates after infection with WNV [28]. Lymphocytes were similarly lowered in 3 replicates of this experiment, suggesting a possible role for this altered lymph population in the divergent disease course of mosquito-sensitized mice.

**Figure 5:**
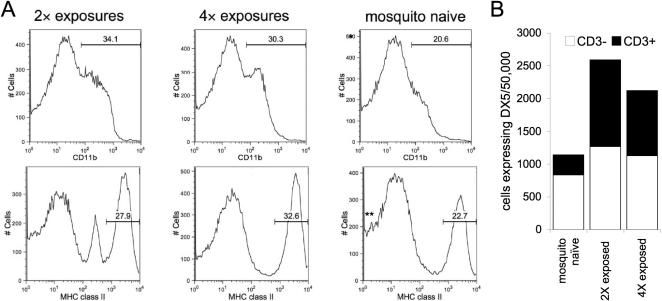
Identification of cell population in draining LN. (A) Histograms representing the quantity of LN cells with a given intensity of CD11b or MHC class II staining at 36 h post infection; the x-axis corresponds to the level of cell surface expression of the indicated membrane protein. The percentage of the LN cell population positive for CD11b or MHC class II is denoted. Asterisks denote a statistically significant difference compared to mice previously naïve to *Ae. aegypti*: * *p*<0.05; ** *p*<0.01. (B) CD3 expression in the DX5^+^ subpopulation of the draining LN of mice 36 h after infection. Groups of mice (n = 3) were exposed 0, 2, or 4 times to mosquito feeding prior to the feeding of a WNV-infected mosquito. Results consist of a pool of cells from three individual mice and are representative of two (B) or three (A) independent replicates.

MHC class II^+^ cells were more abundant in mice previously exposed to mosquitoes (Fig. 5A), making up 27.9% and 32.6% (2 and 4 exposures respectively) of the LN population, juxtaposed with the 22.7% observed in naïve mice (*p*<0.01). Similarly, the LN of 4-times-exposed mice was composed of 30.3% CD11b^+^ cells, as compared to mosquito-naïve mice, for which 20.6% of cells were CD11b^+^ (Fig. 5A; *p*<0.05). Interestingly, cells expressing DX5, a protein associated with natural killer (NK) cells, were found at a higher density in mice previously exposed to mosquitoes (Fig. 5B). Cells dually positive for DX5 and CD3 accounted for the majority of the difference, suggesting an increase NK T in pre-exposed mice.

Additional samples were extracted to determine if differences could be detected during early viral replication. RNA was isolated from the dermal bite site, draining LN, liver, and spleen at 48 h post-infection, and viral load was assessed by real-time RT-PCR. At this early time point only minimal WNV RNA was detectable, with the highest level of virus in the draining LN (Fig. 1B). In most tissues, the level of measurable WNV was too low for robust statistical analysis, but in two of three replicates, the level of virus in the LN was significantly higher in mice previously exposed to mosquito feeding (p<0.01). These data suggest that early expansion of virus is amplified in sensitized mice, a result that is consistent with the increased APC migration to the LN and enhanced IL-10 expression. As expected in mice of this age, viremia was moderate and brief; amongst groups there was no significant difference in virus detected in the serum by TCID_50_ assay (day 3 pi: naïve, 4.01±0.73; pre-exposed, 4.46±0.68 log_10_TCID_50_).

## Discussion

The present research demonstrates that prior exposure to mosquito feeding exacerbates WNV disease. Mice previously-exposed to mosquito feeding develop significantly higher mortality associated with elevation of inflammation, APC recruitment, IL-10 expression in the inoculation site and draining LNs, and LN hyperplasia concurrent with a decrease in lymphocytes chiefly of the CD4^+^ subtype. We suggest the enhanced disease severity is caused by a combination of an increase availability of susceptible cell types, dysregulation of immune signaling, and, potentially, enhanced spread of WNV due to destabilization of tissue integrity mediated by elevated inflammation. Whereas amplified inflammation and cell influx elicited by prior exposure to sand flies [16] or ticks [29] are presumably protective in the response against parasites or bacteria, respectively, similar host responses appear to be detrimental during WNV infection. Such a contradictory outcome is likely due to the distinctiveness of viruses and viral infections as compared to other pathogens. Bacteria and parasites respond to their immediate environment, and the shift from vector to host generally requires phenotypic alterations that may leave these microorganisms more vulnerable during transmission and early infection than a virus. The augmented cell extravasation caused by sensitization to vector feeding, whilst potentially damaging to bacteria or parasites, transports cells that are decidedly susceptible to WNV infection. Additionally, previous research suggests a notable distinction: prior exposure to sand fly feeding enhances IFN-γ production [12], whereas sensitization to *Ae. aegypti* feeding leads to a decrease in IFN-γ levels [15]. Whilst a parallel modulation in IFN-γ could not be demonstrated in the current study, such a decline in this anti-viral effector early during infection would be beneficial to a virus.

It is well established that vector saliva plays a part in the pathogenesis of infections. Previous studies investigating the role of the mosquito vectors on arbovirus infection have only used the single exposure to mosquito saliva required for transmission. This study expands our understanding of WNV infection and subsequent early immune responses, mimicking a natural situation where the host is not na•ve, but rather has been previously exposed to mosquitoes. The most robust responses to mosquito bites are seen in individuals that have limited contact with the specific mosquito species, as naturally-acquired desensitization to mosquito bites has been observed in adults during long-term exposure to the mosquito feeding [30]. Thus, given the aggravated outcome of WNV in sensitized mice, it is plausible that the immune response to mosquito salivary proteins and serological naivety to an arbovirus could play a role in the enhanced susceptibility of recent immigrants into endemic areas. Most human adults are sensitized by more than one mosquito species [30]. Thus cross-reactivity among mosquito species and other insect antigens may enhance the volatility of this effect on subsequent arbovirus infections within individuals, although the effect of cross-reactivity is difficult to predict and requires further investigation.

This research for the first time assesses the pathogenesis of arbovirus infection in hosts immunologically familiar with mosquito feeding, a circumstance which more closely parallels natural transmission. Intriguingly, this is the first report that sensitization to arthropod saliva exacerbates arthropod-borne infection, contrary to previous studies with parasite and bacteria infections transmitted by arthropods. This research demonstrates that not only the seroreactivity of the host to virus is important, but the immune response to vector feeding may also play a role in the infection.[31]

## Methods

### Mice

All animal experiments were performed in accordance within approved Institutional Animal Care and Use Committee (IACUC) guidelines. Four-week old female BALB/c mice (Harlan) were used in all experiments. At least six mice were used for each experimental group, and in those replicates where flow cytometric analysis was performed 10 mice were included in each group. Mice were divided into two main groups that were further sub-divided depending on the requirements of the experiment. The three main groups were: control mice never exposed to mosquitoes or WNV; mice that were previously naïve to mosquito exposure prior to the feeding of an infected mosquito; and mice that were exposed to uninfected mosquitoes before being exposed to a WNV-infected mosquito. Following infection mice were returned to cages, bled periodically, and observed for symptoms of disease. In some experiments a subset of three mice was euthanized at 36 h post infection and tissues isolated for flow cytometric and immunohistochemical analysis.

### Pre-exposure and infection of mice

Mosquitoes used for pre-exposure were uninfected female *Ae. aegypti* 7–10 days post-eclosion that were deprived of sucrose for 24 h to encourage feeding. For pre-exposure, mosquitoes were allowed to feed on the ears (15–20 mosquitoes) of sedated mice for a period of ∼1 h. Mice were exposed once a week for 4 weeks or twice biweekly. Two weeks after the final exposure to uninfected mosquitoes, mice were exposed to WNV via the feeding of a mosquito. Mosquitoes used for infection were infected with WNV [strain 114: GenBank accession nos. AY187013 and AY185907 [32]] by intrathoracic inoculation [33]. Seven days later infected mosquitoes (1 per mouse) were fed on mice. Individual mosquitoes were separated into cartons covered with mesh, mice were sedated and restrained, and the site of feeding was restricted to the ear using a template designed with cardboard and latex. Following feeding each mosquito was tested via real-time RT-PCR [34] to confirm infection status −100% of mosquitoes were positive with equivalent and high viral loads.

### Flow Cytometry

#### Isolation of tissue

Mice were deeply sedated and euthanized via halothane inhalation. Death was assured by cervical dislocation and limbs were immobilized on a dissection board. Ears were surface sterilized with 70% ethanol, tape-stripped by repeated applications of cellophane tape to remove stratum corneum, surgically removed with clean dissection scissors, and placed individually in the wells of a 24-well plate filled with 70% ethanol for 15 min.

After incubating, ears were removed and, using fine forceps, split ventrally along the cartilage into dorsal and ventral ‘leaflets’ and placed into 2 mL of Roswell Park Memorial Institute (RPMI; Sigma-Aldrich) medium supplemented 1:100 with Liberase CI (10 mg/ml; Roche, Indianapolis, Indiana) in 24-well plates for 30 min at 37°C. Following incubation, Ears were removed from medium and placed individually into medicons (Becton Dickinson, Franklin Lakes, New Jersey) with 500 µl of their own medium and run in a Medimachine™ (Becton Dickinson) for 5 minutes. Subsequent to the 5 min processing, 10 ml of DNase medium [RPMI complete (10% FCS with 1∶100 each L-glutamine, penicillin, streptavidin) and 0.05% DNase25 (Sigma, St. Louis, Missouri)] was simultaneously added to the top while being withdrawn from the bottom, effectively flushing the unit. The 10 ml of medium that was extracted from the medicon was then gently forced through a 70 µm-filicon (Becton Dickinson) into a 10-ml conical tube, and cells were kept on ice for remainder of preparation. Cells were then pelleted with a 10 min centrifuge at 1,400 rpm and 4°C. The cell pellet was resuspended in 10 ml of FACS staining buffer (0.09% sodium azide, 1% heat-inactivated fetal calf serum, Dulbecco's PBS without Mg2+ or Ca2+ adjusted to pH 7.4–7.6 and filter through 0.2 µm pore membrane) and pelleted again by centrifugation for 3 min at 4°C and 1,400 rpm. This wash step was completed twice to remove residual non-cellular material, cells were resuspended in 1 ml FACS staining buffer, and a total viable cell count was performed using a hemocytometer and trypan blue (0.4%).

For LN isolation, submandibular LNs draining the ear were teased from surrounding fat and collected in chilled Iscove's modified Eagle's medium (IMEM). Lymph nodes from each mouse within a group were kept separate in some replicates and pooled in others. Lymph node cells were separated using a 70 µm nylon mesh cell strainer. Cells were passed through filter 3 times, then centrifuged at 1,400 rpm and 4°C for 10 minutes. Cells were resuspended in 3 ml FACS staining buffer (0.09% sodium azide, 1% heat-inactivated fetal calf serum, Dulbecco's PBS without Mg2+ or Ca2+ adjusted to pH 7.4–7.6 and filter through 0.2 µm pore membrane) and a total viable cell count was performed using a hemocytometer and trypan blue (0.4%). Cells were stained and analyzed via flow cytometry using standard methods [12], [35].

### Real-time RT-PCR to Detect Shifts in Cytokine mRNA in LN and Skin

To determine whether prior exposure to mosquitoes alters cytokine signaling after initial WNV transmission, real-time RT-PCR was performed on primary sites of WNV replication. Following isolation of cells from ears and LNs, approximately 10^7^ cells per tissue (less for ears) were reserved for RNA isolation. Cells were pelleted by centrifugation and resuspended in 600 µl of RLT lysis buffer (Qiagen, Valencia, California), vortexed for 15 sec, and then RNA was isolated according to manufacturer's protocol. Samples were analyzed for variation in cytokine expression, predominantly assessing IL-2, IL-4, IL-10, IL-12, and IFN-γ mRNA levels. Protocols for running real-time RT-PCR, normalizing between samples, and analyzing results have been previously described [9].

## Supporting Information

Methods S1 (0.03 MB DOC)

Methods S2 (0.03 MB DOC)

Methods S3 (0.03 MB DOC)
