# Supplementary material for: Prior Exposure to Uninfected Mosquitoes Enhances Mortality in Naturally-Transmitted West Nile Virus Infection
Source: PLoS One. 2007 Nov 14;2(11):e1171. doi: 10.1371/journal.pone.0001171 (PMC2048662; doi:10.1371/journal.pone.0001171)
Supplement: Methods S1 — (0.03 MB DOC) [file pone.0001171.s001.doc]

**Supporting Information: Methods S1**

Passive Transfer of Mouse Anti-Mosquito Saliva Antibodies

To assess the role of antibody directed against mosquito saliva in the divergent WNV disease course observed in mosquito naïve and mosquito exposed mice, serum from *Ae. aegypti*-sensitized BALB/c mice was used for passive transfer experiments. Blood was collected from uninfected mice exposed at four weekly intervals to *Ae. aegypti* feeding, and was centrifuged at 500 g for 5 min. Serum samples were pooled, and 200 μl was inoculated undiluted intraperitoneally into mosquito naïve mice. Twelve hours later mice were challenged via the feeding of a single infected mosquito. Vital status was observed twice daily.
