# Supplementary material for: Prior Exposure to Uninfected Mosquitoes Enhances Mortality in Naturally-Transmitted West Nile Virus Infection
Source: PLoS One. 2007 Nov 14;2(11):e1171. doi: 10.1371/journal.pone.0001171 (PMC2048662; doi:10.1371/journal.pone.0001171)
Supplement: Methods S2 — (0.03 MB DOC) [file pone.0001171.s002.doc]

**Supporting Information: Methods S2**

Sodium dodecyl sulfate-polyacrylamide gel electrophoresis (SDS-PAGE) of SGE and Western Blot with mouse serum

To assess the reactivity of mouse sera to mosquito salivary proteins electrophoresis was performed on *Ae. aegypti* SGE for use in Western blots. This was performed on a Mini-PROTEAN II electrophoresis system (Biorad, Hercules, California). A separating gel was prepared in a volume of 30 ml: 7.5 ml 40% acrylamide:Bis (30:1), 15 ml 1.5 M Tris-HCl (pH 8.9), 7.5 ml H2O, 0.3 ml 10% SDS (Biorad), and 0.3 ml 10% NH4S2O8. Five milliliters of this solution was reserved in a separate tube, and 10 μl of N,N,N',N'-tetramethylethylene diamine (TEMED) was mixed into remaining solution and poured between glass plates. After polymerization (5-10 min), 25 μl of TEMED was added to the rest of the separating gel solution, mixed and poured between the glass plates, leaving space for wells and concentrating gel. The concentrating gel was prepared in a 10 ml volume: 1 ml 40% acrylamide:Bis (30:1), 2.5 ml 0.5 M Tris-HCl (pH 6.8), 6.5 ml H2O, 0.1 ml 10% SDS, and 0.1 ml 10% NH4S2O8. Once the separating gel was polymerized, 10 μl of TEMED was added to concentrating gel solution, mixed, poured on top of separating gel, and the comb was inserted. Subsequent to polymerization of concentrating overlay, the comb was removed and the gel was placed onto the gel sandwich clamp assembly, affixed to the inner cooling core, and then into the Mini-PROTEAN II electrophoresis cell lower buffer chamber (Biorad). Both chambers were filled with running buffer, the combs were removed and wells were washed with running buffer. Approximately 30 salivary gland pairs were suspended in 300 μl of PBS then the solution was sonicated and centrifuged at 13,000 rpm for 10 min at 4°C to release salivary proteins and remove residual cellular debris. Protein loading buffer was added (1:4), and the sample and protein markers were heated at 95°C for 5 min. The equivalent of one salivary gland pair was added to each well. Electrophoresis was run at 50 V until the samples/dye lined up at the separating gel, then a constant 20 mA current was set and sample was run until the dye reached the bottom of the gel.

The Western blot was conducted using standard procedures. Transfer was performed in the 4°C refrigerator at 30 V for 1 h. Proteins were fixed on membrane (0.25 g Ponceau C, 0.5 ml HAc, and 50 ml H2O) for 15 min with agitation. Membranes were then fixed several times with Milli-Q water, and the quality of transfer was confirmed. Membranes were blocked (5% dry milk, 1 mg/ml bovine serum albumin, 0.5% Tween20 in PBS; 10 ml per membrane) over-night with agitation at 4°C. Pooled mouse serum (400 μl) was applied to separated regions of the membrane using the Mini-PROTEAN® II Multiscreen Apparatus (Biorad), diluted 1:100 (10% blocking buffer in 0.5% Tween20 in PBS), for 1 h at room temperature. Unbound antibody was then removed with 3 washes (20 mM Tris-HCl pH 7.5, 0.5 M LiCl, and 0.1% Tween20 in H2O) of 10 minutes. The membrane was incubated with the peroxidase-labeled anti-mouse IgG, diluted 1:5000 (10% blocking buffer in 0.5% Tween20 in PBS), for 1 h at room temperature. Unbound secondary antibody was then removed with 3 washes (20 mM Tris-HCl pH 7.5, 0.5 M LiCl, and 0.1% Tween20 in H2O) of 10 minutes. Membranes were treated with ECL plus Western Blotting Detection System (Amersham Biosciences, Piscataway, New Jersey) for 30 sec. The membranes were then placed into an exposure cassette, and Hyperfilm ECL was then exposed to membranes for 0.5 – 2.0 min. Film was developed and serum antibody binding to mosquito salivary proteins assessed.
