# Supplementary material for: Prior Exposure to Uninfected Mosquitoes Enhances Mortality in Naturally-Transmitted West Nile Virus Infection
Source: PLoS One. 2007 Nov 14;2(11):e1171. doi: 10.1371/journal.pone.0001171 (PMC2048662; doi:10.1371/journal.pone.0001171)
Supplement: Methods S3 — (0.03 MB DOC) [file pone.0001171.s003.doc]

**Supporting Information: Methods S3**

Apyrase Assay

To determine whether antibodies against specific mosquito salivary proteins can hinder protein function, an enzymatic assay was used to assess the activity of apyrase, a key mosquito salivary protein [1], in the presence of mouse serum. A stock solution of *Ae. aegypti* SGE was prepared for use throughout assays. Solutions were created by isolating salivary glands from 30 mosquitoes into 300 μl of 0.1 M NaOH. Salivary proteins were solublized and used in the assay at a concentration of 0.1 salivary gland pair/reaction. Reactions were prepared in a 96-well plate and when used, murine serum pools were from groups (exposed or unexposed to *Ae. aegypti*) were diluted 1:20 in buffer, tested in triplicate, and allowed to incubate on the plate with SGE prior to addition of (adenosine triphosphate) ATP. This dilution of serum from mice sensitized to *An. stephensi* was previously shown to cause inhibition of greater than 90% of salivary gland apyrase activity, while titers of 1:160 retained the ability to inhibit more than 50% of apyrase activity [2]. Salivary solutions were added to the plate and to them were added pooled serum, 0.1 NaCl, or nothing. Samples were allowed to incubate with SGE for 30 min. Reaction buffer (0.05 M Tris-HCl pH 9.0, 0.1 M NaCl, 5 mM CaCl2, and 20 mM β-mercaptoethanol added just before reaction) was mixed with ATP stock to give a 2 mM concentration of ATP, and was preheated directly before use to 37°C. One hundred microliters of reaction buffer were added to each well and the plate was incubated at 37°C for 10 minutes. While the incubation was ongoing, PO4 – standards were made with serial dilutions of sodium phosphate stock (1, 0.5, 0.25, 0.125, 0.0625, 0.031 mM). Following the incubation period, standards were added to the plate. To each well was added 25 μl acid molybdate (1.25 g ammonium molybdate dissolved in 100 ml 2.5 N sulphuric acid) and 3 μl reducing mixture (0.2 g 1-amino-2-napthol-4-sulfonic acid, 1.2 g sodium bisulfate, 1.3 g sodium sulfite; this was stored in the dark, and 25 mg was mixed with H_2_O just prior to use), and samples were incubated at 37°C for 20 min. Plates were read with an enzyme-linked immunosorbent assay (ELISA) plate reader at 660 nm. Standards were used to create a line equation that yielded mM of PO4 - from values ELISA values

1. Champagne DE, Smartt CT, Ribeiro JM, James AA (1995) The salivary gland-specific apyrase of the mosquito Aedes aegypti is a member of the 5'-nucleotidase family. Proc Natl Acad Sci U S A 92: 694-698.

2. Mathews GV, Sidjanski S, Vanderberg JP (1996) Inhibition of mosquito salivary gland apyrase activity by antibodies produced in mice immunized by bites of Anopheles stephensi mosquitoes. Am J Trop Med Hyg 55: 417-423.
